# Effectiveness of the Laser Application in Temporomandibular Joint Disorder: A Systematic Review of 1172 Patients

**DOI:** 10.1155/2020/5971032

**Published:** 2020-09-11

**Authors:** Abdalwhab Zwiri, Manal Ahmad Alrawashdeh, Mohammad Khan, Wan Muhamad Amir W. Ahmad, Nur Karyatee Kassim, Jawaad Ahmed Asif, Khoo Suan Phaik, Adam Husein, Zuryati Ab-Ghani

**Affiliations:** ^1^School of Dental Sciences, Universiti Sains Malaysia, Health Campus, 16150 Kubang Kerian, Kota Bharu, Kelantan, Malaysia; ^2^School of Computer Sciences, Universiti Sains Malaysia, 11800 Gelugor, Penang, Malaysia; ^3^Hospital Universiti Sains Malaysia, 16150 Kubang Kerian, Kota Bharu, Kelantan, Malaysia; ^4^Department of Oral Diagnostic and Surgical Sciences, School of Dentistry, International Medical University, 57000 Bukit Jalil, Kuala Lumpur, Malaysia

## Abstract

**Objective:**

The aim of this systematic review was to evaluate the effectiveness of laser application in temporomandibular joint disorder.

**Methods:**

PubMed, SCOPUS, Science Direct, Web of Science, and Google Scholar electronic databases were searched systematically with restricting the languages to only English and year (January 2001 to March 2020), and studies were selected based on the inclusion criteria. Study quality and publication bias were assessed by using the Robvis, a software package of *R* statistical software.

**Results:**

This systematic review included 32 studies (1172 patients) based on the inclusion and exclusion criteria. Most of the studies reported significant reduction of pain by the use of the laser during TMD treatment. Two-thirds of the study (78.13%) found a better outcome comparing with conventional one. According to Robvis, 84.4% of the studies were high methodological studies with low risk of bias.

**Conclusion:**

TMD patients suffer with continuous pain for long time even after conventional treatment. Laser therapy shows a promising outcome of pain reduction for TMD patients. Therefore, laser therapy can be recommended for the TMD patients' better outcome. This trial is registered with PROSPERO (CRD42020177562).

## 1. Introduction

Temporomandibular disorder (TMD) is defined as a series of clinical problems involving muscles of mastication, temporomandibular joints (TMJ), and related structures, identified by facial pain in the TMJ region and masticatory muscle, limited or deviated mandibular movement, and TMJ sounds during jaw movement and action [1]. While they have long been the subject of research, there are still many questions regarding their etiology, diagnosis, and management. Multifactorial TMD etiology is widely established, comprising the involvement of parafunctional behaviors, trauma, stress, and psychological, systemic, genetic, and occlusal causes. None of these variables has proved to outweigh the others, however [2]. The main reason for pain in the orofacial area that does not derive from dental arches is the TMD. In the community, at least one sign is confirmed by 40%–75% of healthy individuals, and at least one symptom of TMD is observed by 33%. 40–75% of healthy individuals in the population have at least one TMD sign, and 33% have at least one TMD symptom. Periarticular tissues (capsule, synovium, and TMJ ligaments), collateral ligaments, and posterior attachments are the most affected anatomical structures of TMJ due to these diseases [3]. Depending on the multifactorial etiology of these problems, the treatment typically requires more than one approach to optimize any potential results, such as medication, behavioral therapy, and physical therapy [2].

In the last few years, laser light has been extensively used in clinical dentistry for the treatment of soft tissue disorders, hypersensitivity of dentine, bone regeneration, and musculoskeletal pain. In TMD patients, LLLT has been used by conservative treatment methods to enhance function and decrease symptoms [3]. The main impacts of laser (LLLT) are biosimulative, regenerative, analgesic, and anti-inflammatory [4]. Helium-neon laser (He-Ne gas) and infrared laser with gallium-arsenium (Ga-As) diode or gallium-aluminum-arsenium (Ga-Al-As) rays are the most common types of laser rays. [5]. The LLLT, known as a soft laser, has low energy intensity and has no effect on skin temperature. The main effect of LLLT is based on the light-absorption process. This soft laser has a wavelength of between 630 nm and 1300 nm [3]. The relative clinical effectiveness of LLLT in treating temporomandibular (TMD) disorders is controversial. Several authors identified LLLT's effectiveness as superior to placebo treatment, while others observed no significant differences between LLLT and placebo for TMJ pain measures, in the view of fact that outcomes in LLLT trials can rely on sample size, population, treatment protocols, and methodology [6, 7].

Therefore, the aim of this systematic review was to find out the effectiveness of laser application in temporomandibular joint pain and review the evidence from previous studies with their sample size and methodology in the management of TMD. This review will provide a precise and obvious knowledge about the benefits and procedures of laser application, which have already been successfully established in TMD management.

## 2. Materials and Methods

### 2.1. Search Strategy

**Figure 1:**
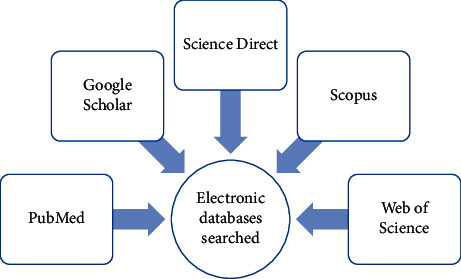
Five electronic databases searched for this review.

Articles were searched in five electronic databases (Figure 1) where the following keyword combinations were used: TMJD + laser, TMJ problem + laser, TMD + laser, TMJD/TMD management + laser application, and TMJ + laser application. Articles between the year 2001 and 2020 were reviewed and systematically searched for those literature published until March 2020. After final screening, total of 32 articles were included in this systematic review. The search encompasses articles (full text) written in English and published in peer-reviewed journals related to TMJ diseases where laser application was performed in different levels.

### 2.2. Study Selection

Here, the prime concern was to find out the uses of laser in temporomandibular disorder patients in terms of reduction of pain and tenderness, improvement of mouth opening and joint sounds, and improvement in the range of jaw motion. The criteria for inclusion have been established as papers using the search keywords mainly TMJ problems and laser application. At the other side, the papers that use laser in TMJ diseases along with other problems such as anatomical defects, anomalies, myofascial pain disorder syndrome (MPDS), soft and hard tissue pathology (tumor, cancer), and previous record of TMJ surgery were excluded from the study. In the context of the exclusion criteria, it also added that those studies were not conducted in human (such as animal studies), and publications in other languages beside English were excluded. The case reports and letter to editor were also excluded from this review. Titles and abstracts of identified studies were assessed independently to judge if the studies match the inclusion criteria.

### 2.3. Data Extraction and Organization

Data were extracted based on the first author, year of publication, number of samples, age and gender of samples, types of TMJ problem, laser types, laser energy and application rate, and results. The data were extracted and double-checked by the authors.

## 3. Results

### 3.1. Selection of Studies

**Figure 2:**
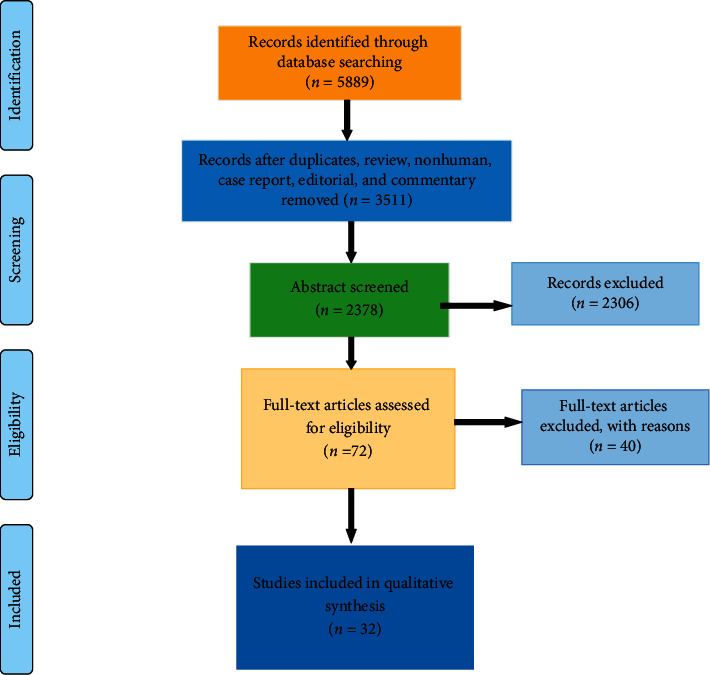
PRISMA flow chart diagram of search strategy and selection of the studies.

At the beginning, this research search strategy provided a total of 5889 papers from databases such as PubMed, Web of Science, Google Scholar, SCOPUS, and ScienceDirect. The remaining 2378 papers were further screened after eliminating 3511 papers in the detection phase (nonhuman topics, summary documents, case reports, editorials, letters and comments, and duplicate studies). A total of 72 studies were considered worthy, but due to unusable data format, forty studies were excluded. Thus, eventually, based on the research goals and inclusion and exclusion requirements, 32 studies (1172 TMD patients in total) were included in this study (Figure 2), and the full text of all the included studies has been retrieved.

### 3.2. Study Characteristics

The key characteristics of the included studies are presented in Table 1. All the studies included were journal articles and most are adults. Among these 32 studies, thirteen were conducted in Brazil, five in European continent and Iran, respectively, three from India, two from Turkey and Taiwan, and one from Malaysia and Iraq. Ga-Al-As (LLLT) laser with a variation of 780–904 nm wavelength is used in most of the studies to treat the TMD patients. Twenty-five studies reported better outcome by reduction of TMD pain compared with conventional treatment modalities, while 7 studies did not find any significant difference between conventional and laser treatment.

### 3.3. Risk of Bias Assessment

**Figure 3:**
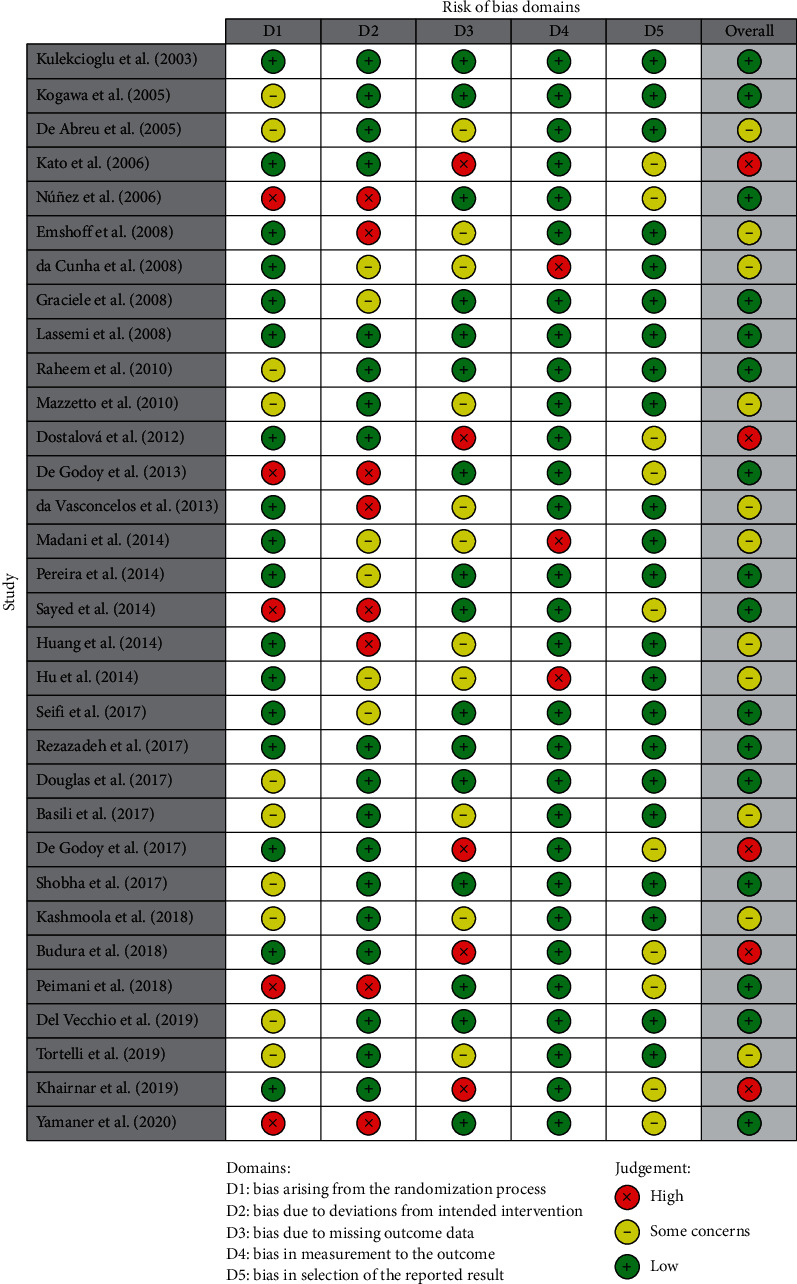
Risk of bias assessment of the study.

Publication bias was assessed by using *R*-based Robvis software package introduced by the National Institute for Health Research (NIHR) [36]. Based on visual inspection of the figure, there is no protentional publication bias in this study assessing the effectiveness of laser treatment for TMD patients (Figure 3). Out of 32 studies, twenty-seven (84.4%) are high methodological studies, which have an overall low risk of bias with some concerns, while only 5 studies have only high risk of bias.

## 4. Discussion

TMD management is very complex and contentious due to the difficulty of determining the exact reasons of the disease and its multifactorial character. The severity varies greatly, and the procedure is varying in terms of duration and invasiveness. Nevertheless, TMD treatment is intended to minimize discomfort, enhance mobility, and delay the progression of internal derangement as accepted by the American Society of Temporomandibular Joint Surgeons guidelines [1]. TMD has a multifactorial nature or etiology; therefore, it is very difficult to get the desired treatment outcome, especially for those patients experiencing severe pain and limited jaw movements.

However, this systematic review tried to overview the role of laser in the management of TMJ disorder patients. After completing this review, the result showed a huge role of laser in TMD management. Most of the studies used LLLT for management of TMD, where it showed a tremendous action in reducing pain, joint clicking, muscle tenderness, and jaw movements.

A study was conducted by Kulekcioglu et al. in 2003 among Turkish population to investigate the effectiveness of low-level laser therapy in the treatment of TMD. Results of the study showed a significant reduction of pain and improvement in maximum mouth opening, lateral motion, and number of tenderness points. According to Kulekcioglu et al. , LLLT in treating TMD may be considered as an alternative physical modality [8]. Another study by Kogawa et al. also found an increase in maximum mouth opening and a decrease in tenderness to palpation in TMD patient after receiving LLLT. Author recommended that LLLT was effective in the management of myogenic TMD [9].

On the other hand, some researchers also tried to find out the effectiveness of the laser treatment in TMD patient, and they concluded with no significant role of LLLT. In 2005, Abreu et al. conducted a study to assess the efficacy of low-intensity laser therapy (LILT) in temporomandibular joint (TMJ) pain and mandibular dysfunction patients. The study had 2 groups, placebo and experimental (LILT). They used the infrared laser (780 nm, 30 mW, 10 s, and 6.3 J/cm2) at three TMJ points. Even though the patient treated with laser had good pain reduction, the result showed no significant changes between placebo and laser groups. Therefore, the researcher did not recommend infrared LILT as a better treatment option. Though there are benefits of applying laser in TMDs management because of noninvasiveness and cost efficient, it has no reported side effects [10]. Another research was performed by Emshoff et al. in Austrian population to evaluate the effectiveness of LLLT in TMJ pain management. A red-beam laser (Model 2000; Helbo Medizintechnik, Austria) (632.8 nm HeNe laser, continuous wave, 30 mW output power, 1.5 J/cm2 energy density) was used. They used three follow-ups from baseline to measuring the visual analogue scale (VAS). The results also presented no significant changes in the management of TMJ. The study recommends that LLLT is no better at minimizing TMJ pain during action than placebo [7]. Similarly, da Cunha et al. reported no significant difference between placebo and laser groups [12]. Authors used Ga-Al-As (gallium-aluminium-arsenide) low-level laser (Biolux laser – Bio-Art, São) with 830 nm wavelength and an output of 500 mW for 20 sec. This study used craniomandibular index (CMI) and VAS for measuring effectiveness of treatment, which results in no significant difference in 2 different protocols, while the patient treated with LLLT reported better pain reduction [12].

Although some authors did not notice any important differences, some studies showed better results when comparing the LLLT with a placebo control group. In 2010, Raheem et al. observed that LLLT plays a significant role in TMDs management by reducing pain and improving maximum mouth opening, lateral motion, and muscle tenderness. Raheem et al. advised LLLT as an effective therapeutic option in myofascial pain dysfunction of TMJ for its analgesic and functional improvement [5].

Even though LLLT is a type of treatment widely applied in physiotherapy of musculoskeletal disorders, there are only some studies that discuss its use in the management of TMD. In 2012, Dostalová et al. performed a research to observe the activity of TMJ and its surrounding tissues and compared the objective results of the effect of LLLT. LLLT was beneficial in the progress of the range of TMD and facilitated a significant pain symptoms reduction [16]. According to Catao et al. , laser therapy was very effective in the pain control and mouth opening of TMDs patients [18]. A study conducted by Sayed et al. confirmed LLLT with satisfactory outcome reducing the pain intensity, number of tender points, joint sounds, and improvement in the range of jaw movement. Therefore, it is an effective and efficient method for treating TMDs [21].

Few more studies have been performed in 2017 by several researchers to evaluate the effectiveness of laser therapy in the treatment of TMDs. Based on the sample size, population, and study design, the result showed some controversy about laser treatment. In 2017, Rezazadeh et al. examined 45 Iran patients to discover the effectiveness of transcutaneous electrical nerve stimulation (TENS) and LLLT in treatment of TMD patients who did not respond to pharmacological therapy. The result showed a significant reduction of pain and tenderness in TMD patients [25]. Another study was performed by Seifi et al. in 40 patients of Iran to assess the result of low-level laser (LLL) therapy and transcutaneous electric nerve stimulation (TENS) on TMDs. The author suggested that TENS or LLL therapy may be useful in improving TMD symptoms at least for the short term [24]. de Godoy et al. carried out a study using a wavelength of 780 nm, energy density of 25 J/cm^2^, power of 50 mW, power density of 1.25 W/cm^2^, and a 20-second exposure. He did not find any significant differences after laser application [28]. This may be the use of the measurement tool used in the study. They compared the difference with the help of electromyography (EMG) signal, while VAS is more specific, accurate, and widely used for pain assessment. Similarly, Shobha et al. conducted a study using 8 sessions of active LILT with a specific diode laser (gallium-aluminum-arsenide, 810 nm, 0.1 W), while the most commonly used therapeutic laser in laser research has been the Ga-As-Al, a semiconductor laser. The laser group showed better improvement in pain reduction even after the 1-month follow-up compared to the placebo group in the VAS score, having no overall significant differences after receiving LLLT [29].

Though, clinically, the use of LLLT is a better procedure in managing TMJ pain. In 2018, a study completed by Buduru et al. showed a significant pain reduction and noticed that there is no disadvantage of LLLT. Thus, the author had recommended the use of LLLT for pain reduction in TMD patients [30]. According to Del Vecchio et al. , LLLT can significantly reduce TMD pain symptom, and it is very much effective in TMJD pain management (Del Vecchio et al. ). Another study performed by Khairnar et al. also found a significant reduction of TMDs pain with LLLT. That study recommends LLLT for treating TMD-related pain with no underlying bony pathology [34].

In the present year 2020, a study was conducted by Yamaner et al. in Turkey to investigate the impact of the ozone and low-level laser (LLL) therapies on pain and function in TMDs patients with disc displacement with reduction. The results of the study support the application of ozone as an effective therapeutic tool for pain relief and LLL as a supportive therapy for temporomandibular disorders [35]. In this systematic review, the author tried to investigate the effectiveness of laser application in temporomandibular joint pain. However, the goal of this systematic review has been achieved. From the above discussion, it is clear that the use of laser in TMD patient is controversial because of its positive and negative outcomes in several studies. But after this review, it can be clearly suggested that the use of the laser has been recommended by most of the researchers. Laser application plays an effective and potential role in the treatment of TMDs patients.

Although the present study went through a systematic search strategy and review of the selective articles, one of the limitations of the present study was the database searching. Due to the limited access of database, the author only searched in five specific databases. This study advised to perform another systematic review with meta-analysis by including some more databases searching to strengthen the findings.

## 5. Conclusion

TMD patients mostly suffer with pain symptoms along with other problems. Nowadays, LLLT became very popular because of its effective role in pain reduction and no known side effects. This systematic review evaluated the effectiveness of the laser application in TMD patient by thorough investigation of the previous studies that have been conducted on laser. After this systematic review, LLLT can be recommended as a beneficial treatment approach for TMD patients.

## Figures and Tables

**Table 1:** Major key characteristics of the included studies in this systematic review.

Author and year	Study population	Sample size	Study design	Age/gender	Problem	Laser type	Energy and application rate	Results
Kulekcioglu et al., 2003 [8]	Turkey	35 patients	RCT	20–59 years (female = 28, male = 7)	Orofacial pain, TMJ sounds, limited mouth opening, or TMJ locking	Ga-Al-As (LLLT), 904 nm wavelength	180 seconds, dosage: 3 J/cm^2^ (15 sessions)	Significant reduction of pain and improvement in maximum mouth opening and lateral motion
Kogawa et al., 2005 [9]	Brazil	19 patients	RCT	Mean age, 26.4 years (female = 19, male = 0)	Temporomandibular disorders (TMDs)	Ga-Al-As (LLLT), wavelength of 830–904 nm	4 J/cm^2^ (3 times a week, 10 sessions)	Significant increase in maximum mouth opening and a decrease in tenderness
Abreu Venancio et al., 2005 [10]	Brazil	30 patients	RCT	Not given	Temporomandibular disorders (TMDs)	Ga-Al-As (LILT), 780 nm wavelength	6.3 J/cm^2^ (twice a week for 3 weeks, 6 sessions)	No significant changes
Kato et al., 2006 [2]	Brazil	18 patients	RCT	Mean age, 25.6 years	Temporomandibular disorders (TMDs)	LLLT, wavelength of 830–904 nm	4 J/cm^2^ energy density (10 sessions, 3 times a week for 4 weeks)	Significant decrease in pain and improvement in muscle tenderness
Núñez et al., 2006 [11]	Brazil	10 patients	Non-RCT clinical trials	18–56 years (female = 8, male = 2)	Temporomandibular disorders (TMDs)	Ga-Al-As (LILT), 670 nm wavelength	3 J per site, total time 8 minutes	Significant improvement in mouth opening
Emshoff et al., 2008 [7]	Austria	52 patients	RCT	18 to 58 years	Patients with unilateral TMJ pain	Red-beam laser, 632.8 nm HeNe laser	1.5 J/cm^2^ energy density (2 to 3 treatments per week for 8 weeks)	No significant differences in reducing pain
Cunha et al., 2008 [12]	Brazil	40 patients	RCT	(Female = 39, male = 1)	Temporomandibular disorders (TMDs)	Ga-Al-As (LLL), 830 nm wavelength	20 seconds, 100 J/cm^2^ (once a week for 4 weeks)	No significant changes
Graciele Carrasco et al., 2008 [13]	Brazil	14 patients	RCT	Not given	Temporomandibular dysfunction (TMD)	Ga-Al-As (LILT), 780 nm wavelength	60 seconds, 105 J/cm^2^ (twice per week for 4 weeks)	Significant improvement of masticatory efficiency
Lassemi , 2008 [14]	Iran	48 patients	RCT	(Female = 24, male = 24)	Temporomandibular disorders (TMDs)	Ga-As (LLLT), 980 nm wavelength	2 J per point (2 sessions with a 48-h interval)	Significant reduction of pain severity and clicking
Raheem et al., 2010 [5]	Iraq	34 patients	Non-RCT, convenience sampling	(Female = 21, male = 13)	Temporomandibular disorders (TMDs)	Semiconductor galium-aluminium (gas) LLLT, 785 nm wavelength	Energy density of 16 J/cm^2^ (twice to thrice weekly and repeated 4 weeks, total 10 sessions)	Significant reduction of pain and improvement in maximum mouth opening, lateral motion, and muscle tenderness
Mazzetto et al., 2010 [15]	Brazil	40 patients	RCT	Not given	Temporomandibular disorders (TMDs)	Ga-Al-As (LLL), 830 nm wavelength	10 s, 5 J/cm^2^	Significant improvement in pain reduction and mandibular movement
Dostalová et al., 2012 [16]	Prague	27 patients	Non-RCT	Mean age of male 18.57 and female 27.57 years	Temporomandibular disorders (TMDs)	Ga-Al-As (LLL), 830 nm wavelength	15 s, 4 J/cm^2^ (once a week for 5 weeks)	Significant reduction of pain
De Godoy et al., 2013 [17]	Brazil	85 patients	RCT	15 and 18 years	Temporomandibular disorders (TMDs)	Ga-Al-As (LLL), 780 nm wavelength	20 s, 25 J/cm^2^ (6 weeks, a total of 12 sessions)	Not significant
Catão et al., 2013 [18]	Brazil	20 patients	RCT	19 to 58 years (female = 18, male = 2)	Temporomandibular disorders (TMDs)	As-Ga-Al laser (830 nm wavelength), InGaAIP laser (830 nm wavelength)	4 J/cm^2^ (three times a week for 4 weeks, 12 sessions)	Significant reduction in pain and improvement in mouth opening
Madani *e*t al., 2014 [19]	Iran	20 patients	RCT	35–60 years (female = 19, male = 1)	TMJ osteoarthritis	LLLT (low level laser), 810 nm wavelength	6 J per point, 3.4 J/cm^2^ (three times a week for 4 weeks)	No significant differences (for reducing pain and improving mouth opening)
Pereira et al., 2014 [20]	Brazil	19 patients	RCT	21–55 years (female = 15, male = 4)	Temporomandibular disorders (TMDs)	LLLT, wavelength: 660 nm (red laser) and 795 nm (infrared laser)	4 J/cm^2^ (an interval of 48 hours, total 3 sessions)	Statistically significant in the treatment and remission of TMD symptoms
Sayed et al., 2014 [21]	India	20 patients	RCT	19–47 years (female = 9, male = 11)	Temporomandibular disorders (TMDs)	LLLT semiconductive (diodic) gallium arsenide (GaAs) laser, 904 nm wavelength	60 s, 4 J/cm^2^ (3 times a week for 2 weeks)	Statistically significant in reducing the pain intensity, tenderness, joint sounds, and improvement in the range of jaw motion
Huang et al., 2014 [22]	Taiwan	20 patients	RCT	Not given	Temporomandibular joint disorders (TMDs)	LLLT, 800 nm wavelength	100.5 J/cm^2^ (once a week)	Significant reduction of pain
Hu et al., 2014 [23]	Taiwan	29 patients	Retrospective convenience	17–67 years (female = 25, male = 4)	Temporomandibular disorders (TMDs)	Ga-Al-As (LLL), 810 nm wavelength	5s, 0.375 J/cm^2^ (3 times per week for 4 weeks)	Significant improvement of treatment-resistant TMD
Seifi et al., 2017 [24]	Iran	40 patients	RCT	18–50 years	Temporomandibular joint disorders (TMDs)	Ga-Al-As (LLL), 810 nm wavelength	(Four half-hour sessions per week)	Significant decrease in pain and tenderness
Rezazadeh et al., 2017 [25]	Iran	45 patients	RCT	Not given	Temporomandibular disorders (TMDs)	Ga-Al-As (LLL), 980 nm wavelength	2.5 minutes, 5 J/cm^2^ (8 sessions within 2 weeks)	Significant reduction of pain and tenderness
Douglas de Oliveira et al., 2017 [26]	Brazil	19 patients	RCT	21–55 years (female = 15, male = 4)	Temporomandibular disorders (TMDs)	Ga-Al-As (LLL), wavelength: 660 nm (red laser) and 790 nm (infrared laser)	1.06 s, 8 J/cm^2^ (3 sessions)	Statistically significant in the treatment of TMD
Basili 2017 [27]	Italy	180 patients	Non-RCT	Not given	Temporomandibular disorders (TMDs)	LLLT, 830 nm wavelength	3 sessions	Significant reduction of pain
De Godoy et al., 2017 [28]	Brazil	16 patients	RCT	14–23 years	Temporomandibular disorders (TMDs)	LLLT, 780 nm wavelength	20 s, 25 J/cm^2^ (12 sessions)	No significant changes
Shobha et al., 2017 [29]	India	40 patients	RCT	18–40 years	Temporomandibular disorders (TMDs)	Ga-Al-As (LLLT), 810 nm wavelength	60 s, 6 J/cm^2^ (2-3 times a week, 8 sessions)	No significant changes
Kashmoola 2018 [1]	Malaysia	22 patients	Non-RCT	18–68 years	Temporomandibular disorders (TMDs)	LLLT	2-3 minutes, 0.5 W, 30 Hz daily for 3 days and then once a week for 2 weeks	Significant reduction of pain
Buduru et al., 2018 [30]	Romania	20 patients	Non-RCT	Not given	Temporomandibular joint disorders (TMDs)	LLLT, 660 nm wavelength	Energy intensity 90 mW (once each day, five days per week, for a total of 10 sessions	Significant reduction of pain
Peimani et al., 2018 [31]	Iran	72 patients	RCT	20–45 years	TMJ dysfunction	LLLT, 808 nm wavelength	144 J/cm^2^ (2 times a week for 4 weeks)	Significant reduction of pain, clicking, and tenderness
Del Vecchio et al., 2019 [32]	Italy	90 patients	RCT	18–73 years (female = 78, male = 12)	Temporomandibular joint disorders- (TMJDs-) related pain	LLLT, 808 nm wavelength	5 J/min (twice a day for 7 days)	Significant reduction of pain
Tortelli et al., 2019 [33]	Brazil	12 patients	RCT	23–50 years	Temporomandibular disorders (TMDs)	LLLT, 808 nm ± 10 nm wavelength	2 J, (72h intervals, for a total of 6 sessions)	Significant decrease in pain and improve maximal opening capacity
Khairnar et al., 2019 [34]	India	42 patients	RCT	25–45 years	Temporomandibular joint disorders (TMDs)	LLLT, 660 nm wavelength	2.2 J/min	Significant role in treating TMD-related pain
Yamaner et al., 2020 [35]	Turkey	62 patients	RCT	Mean age, 31.51 ± 10.32 years (female = 59, male = 3)	Temporomandibular disorders (TMDs)	LLLT, 820 nm wavelength	10 s, 3 J/cm^2^ (3 times a week, total 6 sessions)	Significant reduction of pain
